# The vesicle trafficking regulator *PN_SCD1* is demethylated and overexpressed in florets of apomictic *Paspalum notatum* genotypes

**DOI:** 10.1038/s41598-018-21220-4

**Published:** 2018-02-14

**Authors:** Marika Bocchini, Giulio Galla, Fulvio Pupilli, Michele Bellucci, Gianni Barcaccia, Juan Pablo A. Ortiz, Silvina C. Pessino, Emidio Albertini

**Affiliations:** 1 Department of Agricultural, Food and Environmental Sciences, University of Perugia, Perugia, 06121 Italy; 2 Laboratory of Genomics, Department of Agriculture Food Natural resources Animals and Environment (DAFNAE), University of Padova, 35020 Legnaro, PD Italy; 3 Institute of Biosciences and Bioresources, Research Division of Perugia, National Research Council (CNR), via della Madonna Alta 130, 06128 Perugia, Italy; 4 Laboratorio de Biología Molecular, Instituto de Investigaciones en Ciencias Agrarias de Rosario (IICAR) CONICET-UNR, Facultad de Ciencias Agrarias, Universidad Nacional de Rosario, Zavalla, S2125ZAA Argentina

## Abstract

Apomixis (asexual reproduction through seeds) is considered a deviation of the sexual reproductive pathway leading to the development of clonal progenies genetically identical to the mother plant. Here we used the Methylation-Sensitive Amplification Polymorphism (MSAP) technique to characterize cytosine methylation patterns occurring in florets of sexual and aposporous *Paspalum notatum* genotypes, in order to identify epigenetically-controlled genes putatively involved in apomixis development. From twelve polymorphic MSAP-derived sequences, one (PN_6.6, later renamed *PN_SCD1*) was selected due to its relevant annotation and differential representation in apomictic and sexual floral transcriptome libraries. *PN_SCD1* encodes the DENN domain/WD repeat-containing protein SCD1, which interacts with RAB GTPases- and/or MAPKs to promote specialized cell division, functions in clathrin-mediated membrane transport and acts as potential substrate receptor of CUL4 E3 ubiquitin ligases. Quantitative RT-PCR and comparative RNAseq analyses of laser microdissected nucellar cells confirmed *PN_SCD1* upregulation in florets of apomictic plants and revealed that overexpression takes place just before the onset of apospory initials. Moreover, we found that several SCD1 molecular partners are expressed in *P*. *notatum* florets and upregulated in apomictic plants. Our results disclosed a specific vesicle trafficking molecular pathway epigenetically modulated during apomixis.

## Introduction

Seed formation in flowering plants can follow variable mechanisms, which have evolved over millions of years to secure persistence and diffusion of adapted genotypes. In this regard, the sexual pathway is far from being the only possible operative route. Seeds can be formed through alternative asexual mechanisms, which are classified under the general term ‘apomixis’^1^. These asexual reproductive pathways lead to the formation of a clonal progeny genetically identical to the mother plant. Apomixis was described in more than 400 flowering plant species belonging to 35 families^2^, but it is absent from gymnosperms^3^, with the exception of a male-type odd case described in *Cupressus*^4^. Despite the complexity and diversity of these alternative reproductive routes, it is widely accepted that apomictic behaviours can be regarded as deregulations of sexual developmental processes in time and space, involving both genetic and epigenetic mechanisms^5^. Often, sexuality and apomixis coexist in the same organism, a condition known as facultative apomixis^1^.

Apomictic developmental pathways are subclassified into two different subcategories: sporophytic and gametophytic^1^. In sporophytic apomicts, somatic (2n) cells from the ovule nucellus or the integuments differentiate to generate multiple globular-shaped embryos, which coexist with the legitimate sexually-formed sibling and share its endosperm for nutrition^6^. In gametophytic apomicts, one or several unreduced (2n) embryo sacs are formed in the ovule nucellus after a series of mitosis^6^. Gametophytic apomixis is further classified into diplospory and apospory, depending on the cell giving origin to the unreduced embryo sacs. They originate from the megaspore mother cell itself in diplosporous plants and from somatic nucellar companion cells in aposporous ones. Embryo development is fertilization independent *i.e*. embryos arise through parthenogenesis, whereas endosperm formation may or may not require fertilization^6^.

Apomixis represents a useful tool for agriculture, due to its intrinsic capacity to immediately fix heterotic combinations^7^. The absence of female meiosis and subsequent egg cell fertilization leads to the emergence of clonal progenies from genetically maternal seeds. However, when used as male parents in experimental crosses with sexual plants, apomictic individuals are able to transfer the trait and produce hybrid progenies segregating for the mode of reproduction. Natural apomictic species, like perennial forage grasses included in the genera *Brachiaria* and *Paspalum*, are currently being improved through novel apomixis-based breeding mechanisms^8–12^. These programs have accelerated the production of adapted hybrid cultivars and are rapidly boosting livestock farming in areas that had traditionally been marginal for cattle production. Harnessing apomixis into major crops like maize, sorghum or rice has proved to be more difficult, since projects aimed at introgressing the trait from wild relatives through sexual crosses were unsuccessful so far^13^. Genetic engineering could provide a possible way out, but the genetic determinants that control the expression of the trait remain unknown. Therefore, a full characterization of the molecular pathways governing apomixis is crucial to overcome barriers banning introduction from wild relatives and/or to allow the modulation of the trait through genetic engineering^14^. Moreover, a deep knowledge of the apomixis genetic determinants will allow a better prediction, avoidance and/or control of potential environmental risks derived from its use in agriculture^7^.

Evidence originated from sexual model species indicated that epigenetic mechanisms are involved in the expression of apomixis, since the disruption of RdDM (RNA-directed DNA methylation) pathways operating in ovules promoted the appearance of phenotypes mimicking the trait. In *Arabidopsis* plants, a loss of function of an ARGONAUTE (AGO) protein family member, AGO9, results in the development of supernumerary unreduced megaspores in the ovular nucellus^15^. In wild-type ovules, the AGO9 protein is not localized in the nucellar gametic cells, but in nucellus layer 1 (L_1_) cells^15^. Since AGO9 is involved in the silencing mechanisms mediated by small RNAs, these data suggest the existence of trans-acting small interference RNA (tasiRNA) pathways operating in the nucellus in order to stipulate the legitimate female gametophytic lineage^15^. Moreover, *ago104* defective maize mutants produce up to 70% of functional unreduced female gametes^16^. Such unreduced gametes arise from mitosis-like divisions of the megaspore mother cells themselves. Thus, whereas *AGO9* mutations produce phenotypes resembling apospory, *AGO104* disruptions ends up in phenotypes mimicking diplospory^15,16^. Several other epigenetic regulators were identified in differential expression analyses of sexual maize and apomictic maize-*Tripsacum* hybrids, including several DNA methyltransferases (*DMT102*, *DMT103* and *DMT105*), a SWI2/SNF2-like chromatin remodeler (*CHR106*), a plant-specific histone deacetylase (*HDT104*) and a histone H1 linker (*HON101*)^17^. Further characterization of *DMT102* and *DMT103* revealed that they are both expressed in germ cells as well as surrounding nucellar cells, and that maize mutants are able to form unreduced gametes and/or extra embryo sacs, and show a release of repressive chromatin states^17^.

*Paspalum* became a model genus for the analysis of aposporous apomixis mechanisms, due to its dual nature as a useful system for mining candidate gene(s) and an important target crop^18^. A wealth of information has been produced regarding the biology, genetic and reproductive modes of many *Paspalum* species including: (1) analysis of cytoembryological aspects of sexual and asexual development; (2) detailed molecular mapping of apomixis loci; (3) availability of candidate genes for the control of apospory; and (4) development of transformation systems for gene delivery^18^. The best-characterized species within the genus are *Paspalum notatum* and *Paspalum simplex*, both represented by two major cytotypes: sexual diploids and aposporous pseudogamous apomictic tetraploids. In both species, aposporous apomixis is controlled by the single genomic superlocus named Apomixis Controlling Locus (ACL), which is blocked in terms of recombination and shows synteny with a subtelomeric region of the rice chromosome 12 long arm, and for *P*. *notatum* only, also with segments of rice chromosome 2 long arm^19–22^. In the particular case of *P*. *notatum*, the ACL also includes segments syntenic to the rice chromosome 2 long arm^19–22^. Partial sequence analysis of the ACL revealed structural features of heterochromatin, namely the presence of repetitive elements, gene degeneration^23^ and deregulation^24^. After examining the cytosine methylation status of the ACL in combination with apomixis-linked BAC-FISH analysis, a high level of methylation was detected^25^. Moreover, DNA methylation is essential for parthenogenesis, since the demethylating agent 5′-azacytidine causes a significant increase of hybrid progenies of a higher ploidy level (BIII hybrids) in the offspring of treated apomictic mother plants^25^.

Investigating the epigenetic-mediated control of asexual development is substantial not only to projects dealing with reproductive biology, but also to apomixis-based breeding programs, since the latter are strongly dependent on the generation of obligate apomictic hybrids with full capacity to form clonal progeny. Due to the highly heterozygous, polyploid and poorly characterized nature of the genome of natural apomictic grasses^22^, the technique chosen to detect epigenetic marks must be independent from any prior sequence information, particularly if a genome-wide analysis is required. The Methylation-Sensitive Amplified Polymorphism (MSAP) methodology enables the evaluation of levels and patterns of DNA methylation from an open and broad perspective, without previous sequence knowledge^26^. It is based on the use of isoschizomers *Hpa*II and *Msp*I, a pair of restriction enzymes that recognize the same target site (5′-CCGG-3′) but have different sensitivity to methylated cytosines^26^. Although revealing only a fraction of the global methylation changes, MSAP can be very useful to investigate epigenetic variations occurring between different genotypes, since differentially methylated sites lacking genetic polymorphisms can be easily discriminated^27^. Among many other applications, MSAP has already been used to study methylation changes during ploidy level conversions in apomictic and sexual *Eragrostis curvula*^28^ and to estimate the cytosine methylation pattern frequencies and variation in diploid and tetraploid cytotypes of sexual and apomictic *P*. *notatum*^29^.

The objective of this research was to detect and validate epigenetically-controlled apomixis candidate genes. Through the use of MSAP, we found that the vesicle trafficking regulator *PN_SCD1* displays gene body hypomethylation and transcript upregulation in the ovule nucellus of *Paspalum* apomictic plants just before the onset of apospory. Moreover, several *PN_SCD1* biological partners showed de-regulated expression in apomictic genotypes.

## Results

### Methylation-sensitive amplified polymorphism analysis

**Figure 1:**
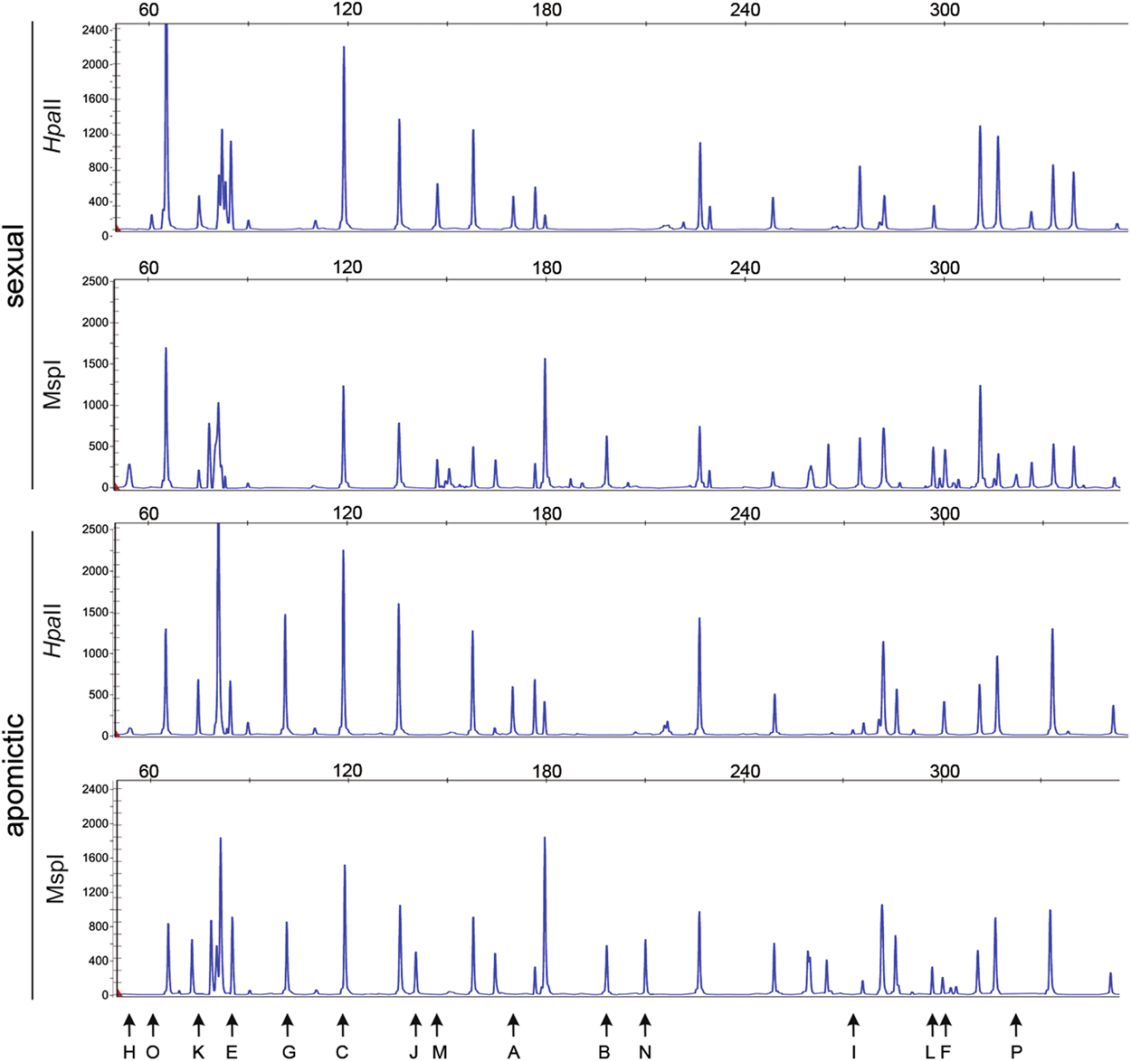
MSAP output and DNA methylation patterns. Methylation patterns corresponding to a single sexual and a single apomictic individual are shown as an example. Arrows and letters indicate each DNA methylation pattern^55^. Sexual and apomictic samples were digested with either *Hpa*II or *Msp*I. Patterns A–C represent monomorphic classes, in which the methylation pattern is the same in the sexual and the apomictic genotype. Patterns E–P are indicative of possible cytosine methylation alterations in the apomictic plant with respect to the sexual one.

**Figure 2:**
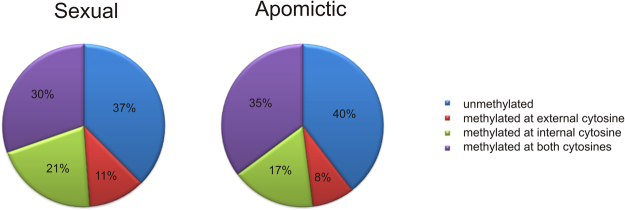
Average proportion of methylation patterns in flowers of sexual and apomictic *P. notatum* genotypes. Both plant types (apomictic and sexual) showed identical general methylation patterns proportions, as indicated by Newcombe tests^30^.

Twelve MSAP primer combinations were used to compare the cytosine methylation status in genomic DNA samples extracted from florets of four sexual (#36, #58, #76, #83) and five apomictic (#Q4117, #Q4086, #9, #40, #112) *P. notatum* individuals. Overall, these experiments generated 547 clearly detectable and reproducible fragments, which were resolved by capillary electrophoresis linked to an automated detection system. An example of the experimental output is shown in Fig. 1. The frequency of occurrence of the different methylation patterns in the analysed genotypes is shown in Table 1. Sexual and apomictic plants displayed, respectively, an average of: (1) 205 ± 7 and 216 ± 16 unmethylated sites; (2) 62 ± 8 and 46 ± 17 externally hemimethylated sites; (3) 114 ± 8 and 92 ± 13 internally methylated sites; (4) 166 ± 3 and 192 ± 32 methylated at both cytosines or absent sites (the latter due to genetic variation). The average distribution of different methylation patterns in sexual and apomictic samples is summarized in Fig. 2. Tests of Newcombe for 95% confidence intervals of proportions estimations^30^ showed that the different methylation patterns occurred in equivalent ratios in sexual and apomictic genotypes. However, a locus by locus analysis across the total 547 loci revealed significant variation in the methylation status among the different genotypes. Moreover, clustering analysis based on an epigenetic similarity matrix^27^ underlined a higher similarity among sexual plants (Supplementary Fig. S1) (see also Discussion).

**Table 1:** MSAP amplification banding patterns and their frequency of occurrence.

**MSAP PATTERN TYPE** ^**a**^	**EP** ^**b**^		**SEXUAL PLANTS**				**APOMICTIC PLANTS**				
	HpaII	MspI	F_1__36	F_1__58	F_1__76	F_1__83	Q4117	Q4086	F_1__9	F_1__40	F_1__112
I: 11 (unmethylated)			212	196	210	203	217	212	229	191	233
II: 10 (methylated at the external cytosine)			51	66	61	69	39	24	45	70	53
III: 01 (methylated at the internal cytosine)			121	120	107	107	89	72	93	97	109
IV: 00 (methylated at both cytosines)^c^			163	165	169	168	202	239	180	189	152

^a^I, II and III: epigenetically informative patterns

^b^EP: electrophoresis pattern. Black boxes indicate the presence of a peak. Light grey boxes indicate the absence of a peak detected in the alternative isoschizomer run and/or other/s sample/s run/s.

^c^Methylation at both cytosines cannot be differentiated from genetic mutations (i. e. absence of the site in the genome of a particular plant).

### Identification of differentially methylated DNA fragments expressed in florets

A total of 26 genomic DNA fragments showing statistically significant differential methylation between sexual and apomictic samples were detected (Supplementary Fig. S2). Amplicons were then resolved in polyacrylamide gels and twelve candidate fragments (PN_1.7; PN_1.8; PN_2.2; PN_2.10; PN_4.8; PN_4.10; PN_6.5; PN_6.6; PN_7.6; PN_7.7; PN_8.5; PN_8.8) were successfully retrieved from the gels, cloned and sequenced. These sequences were used as queries for *in silico* mapping analyses onto the rice genome and transcriptome surveys onto 454 floral transcriptome libraries of sexual and apomictic *P. notatum*^31^. Rice *in silico* mapping results are displayed in Supplementary Table S1. Two pairs of fragments (PN_1.7/PN_1.8 and PN_7.6/PN_7.7) matched to variants of the same sequence and could have been originated from different alleles. Only one fragment (PN_6.6) mapped with top identity onto a functionally characterized gene (BGIOSGA001363, SCD1-like). The rest of the sequences mapped either onto unknown sequences or functionally uncharacterized genes. Regarding the *Paspalum* floral transcriptome survey, three of the sequences were found expressed in the libraries (PN_2.10, PN_6.6 and PN_8.5). The remaining ones did not find a transcribed match. Therefore, they could be included in promoter, intronic, or intergenic regions, as well as belonged to transcribed regions of genes not expressed in flowers. One of the candidates expressed in flowers (PN_6.6) showed a de-methylated pattern in apomictic plants (11) (with the exception of the Q4117 genotype) and an internally methylated pattern in sexual plants (01) (Table 2). The remaining two candidates (PN_2.10 and PN_8.5) displayed a de-methylated pattern is apomictic plants (11) (with the exception of F1 112 genotype) and a fully-methylated pattern (or absence of the site) in sexual plants (00) (Table 2). The PN_6.6, PN_2.10 and PN_8.5 full sequences were recovered from the 454 floral transcriptome databases^31^. Annotations were determined from BLASTX at NCBI and confirmed by comparison with ontology classification analysis performed by INDEAR (Instituto de Agrobiotecnología de Rosario) during the construction of the 454/Roche floral transcriptome libraries. Two of the sequences corresponded to protein-coding genes (PN_6.6 and PN_8.5) and the remaining one was unknown (PN_2.10) (Table 2).

**Table 2:** Differentially methylated candidates represented in the floral 454/Roche transcriptome libraries of apomictic and sexual *P. notatum* genotypes.

MSAP band	MSAP pattern (APO vs SEX)	454/Roche isotig (GenBank TSA accession number)	Number of reads (454)		Differential expression (P value)	Size in base pairs	Annotation	Annotation statistics (blastx)
			Apo reads	Sex reads				
**PN_2.10**	11 vs 00	apoisotig43669 (GFMI02043693.1) sexisotig40004 (GFNR01040007.1)	2	3	1	386–396	Unknown	—
**PN_6.6**	11 vs 01	apoisotig16740 (GFMI02016795.1) sexisotig13672 (GFNR01013696.1)	91	64	0.02474	3775–4008	DENN domain and WD repeat-containing protein	E-value: 0.0; ID: 97%
**PN_8.5**	11 vs 00	apoisotig10437 (GFMI02010491.1) apoisotig10438 (GFMI02010492.1) sexisotig22289 (GFNR01022308.1)	269	269	0.83156	1131–2653	14–3–3 like protein	E-value: 6 e^−174^; ID: 98%

**Figure 3:**
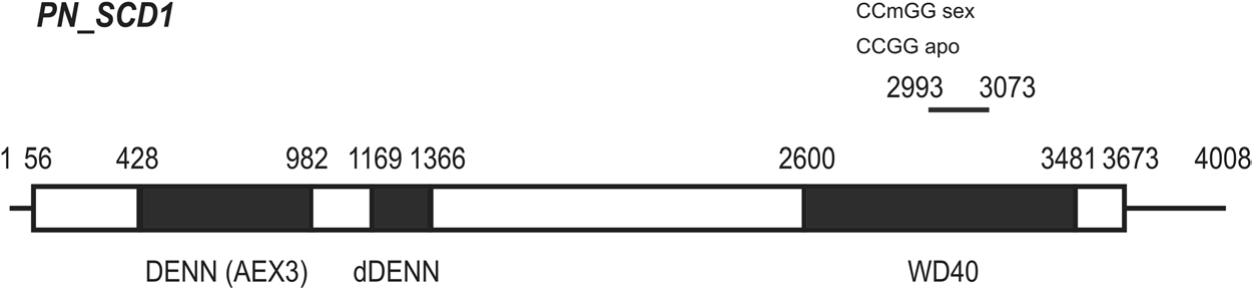
Structural scheme of *PN_SCD1*. The scheme was constructed from sequence of *P. notatum* apoisotig16740 (GFMI02016795.1). White boxes: CDR regions with non-described domains. Black boxes: CDR regions encoding known domains (indicated below). Lines: UTR regions. Numbers indicate the nucleotide positions assigned to each feature. The original fragment isolated from MSAP analysis was represented on top of the scheme, between positions 2993 and 3073. The methylation status of the CCGG site (subjected to MSAP analysis) in apomictic and sexual plants was indicated on top.

One of the sequences (PN_6.6) showed differential expression between sexual and apomictic plants in the 454/Roche libraries (Table 2). NCBI BLASTX searches identified the DENN domain and WD repeat-containing protein SCD1 of *Setaria italica* (XP_004969033.1; E-value = 0.0; ID = 1000/1036, 97%) as the best match for this sequence (Table 2). PN_6.6 was accordingly renamed *PN_SCD1*. The *Arabidopsis* putative ortholog to this gene is At1G49040 (*SCD1*, *STOMATAL CYTOKINESIS-DEFECTIVE 1 SCD1*)^32–34^. The original MSAP fragment mapped onto the WD40 domain of apoisotig16740 GFMI02016795.1, with E-value: 9 e^−30^, ID 99% and 1% gaps (Fig. 3). Due to the relevant annotation of *PN_SCD1* concerning specialized cell division, and the putative differential expression detected in the 454/Roche libraries, we decided to extend the characterization of this particular epigenetic candidate to evaluate its potential as an apomixis associated gene.

### *PN_SCD1* is upregulated in florets of apomictic *P*. *notatum* plants

**Figure 4:**
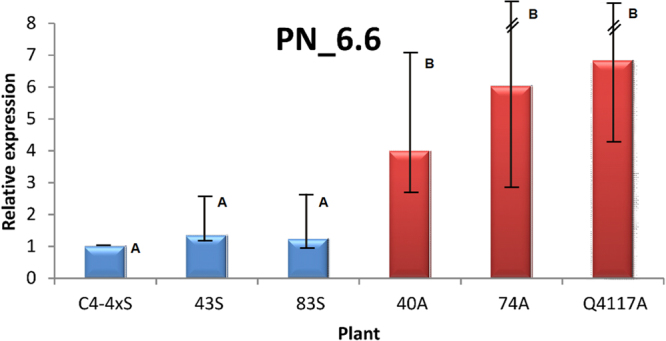
qPCR experiments showed highly significant upregulation of PN_6.6 in flowers of apomictic plants. Comparisons were carried out in three sexual (light blue) and three apomictic (red) plants. Sexual genotype C4-4x was used as control. An expression value = 1 (standard deviation = 0) was automatically assigned to C4-4x by the data processing program (REST RG). The software predicted significant upregulation in apomictic plants, in agreement with the methylation patterns detected by MSAP (Supplementary Table S2). Same letters indicate non-significant statistical differences (overlapping standard errors intervals).

The relative quantitative expression of *PN_SCD1* was investigated in florets collected at anthesis, by conducting qPCR experiments on cDNA samples originated from three sexual (C4-4x, #43, #83) and three apomictic (#40, #74, Q4117) plants. The REST RG relative expression quantitation analysis revealed significant upregulation in all apomictic plants tested. Results are displayed in Fig. 4 and Supplementary Table S2.

### *PN_SCD1* is upregulated in the ovule nucellus of apomictic *P*. *simplex* plants

We investigated the expression in ovules at early developmental stages by using data of *P*. *simplex Illumina* RNAseq laser capture microdissection (LCM) libraries available for sexual and apomictic genotypes. These libraries were constructed from groups of cells of the nucellar tissues of ovules sampled at premeiotic stage in sexual and apomictic plants, just before the differentiation of aposporous initials (Galla G., Barcaccia G., Bellucci M., Pupilli F., unpublished). Although *P. simplex* and *P. notatum* are not closely related within the *Paspalum* genus, they display nearly identical apospory mechanisms (see Discussion). Two *P. simplex* sequences (PS_6.6.1 and PS_6.6.2) scored the best hits with *PN_SCD1* (Table 3). As expected, the similarity parameters pointed to a high degree of sequence conservation with *P. notatum* (Table 3). By using any of the detected *P. simplex* sequences (PS_6.6 0.1 OM_Locus_1_Transcript_365164/578620 or PS_6.6.2 OM_Locus_82107_Transcript_4/4) as query in BLAST searches onto the *P. notatum* apomictic and sexual floral transcriptome libraries, a single match was detected in both the apo libraries (isotig16740, gene = isogroup 06295, length = 4008, num Contigs = 1, E-val 0.0) and the sexual library (isotig13672 gene = isogroup 05214 length = 3775 numContigs = 1, E-value 0.0). Transcript PS_6.6.1 resulted significantly upregulated with 97% confidence in the apomictic LCM libraries (Table 3).

**Table 3:** Quantitative estimations of expression in ovule nucellar cells of sexual and apomictic plants at AI onset stage.

*P. notatum* (query)	*P. simplex* (subject)	Similarity parameters	Size (*P. simplex*)	RPKM (average)^a^		p-value^b^
				APO	SEX	
***PN_SCD1***	PS_6.6 0.1^c^ (OM_Locus_1_Transcript_365164/578620)	E-value: 0.0; ID: 98%; Gaps: 0%	1896	7.62	2.27	0.03386
	PS_6.6.2 OM_Locus_82107_Transcript_4/4	E-value: 0.0; ID: 96%; Gaps: 0%	602	155.43	177.36	0.23311

^a^RPKM = (number of mapped reads)/(length of transcript in kilo bases)/(million mapped reads).

^b^Baggerly’s test for differential expression in apomictic and sexual libraries^58^.

^c^Upregulated at statistically significant level in the ovule nucellus of apomictic plants just before AI differentiation.

### *SCD1* partners’ orthologs are upregulated in florets of apomictic *P. notatum* plants

We controlled the representation of genes related to the SCD1 role as vesicle trafficking regulator and CUL4 E3 ubiquitin ligase substrate receptor in the 454/Roche transcriptome libraries of sexual and apomictic *P. notatum* genotypes. Several candidates were significantly upregulated in apomictic plants (Table 4). Moreover, a PANTHER test showed that biological processes Single Organism Transport (GO:0044765) and Localization (GO:0051179) were overrepresented at highly significant p-values (1.29 E^−06^ and 1.76 E^−07^, respectively) in the list of apomictic vs. sexual differentially expressed candidate genes reported from the *P. notatum* 454/Roche floral transcriptome libraries^31^. These results point to an integral membrane protein turnover and ubiquitination pathway operating specifically during apomixis development.

**Table 4:** Several transcripts associated with *SCD1*-mediated vesicle transport and protein ubiquitination show upregulation in florets of apomictic plants.

Isotig number (apo + sex assembly)	Annotation	Apo reads^a^ (454/Roche)	Sex reads^a^ (454/Roche)	Differential expression^a^ (P-value)
isotig28399	Rab-GTPase-TBC domain (AT3G55020)	85	55	0.00878
isotig27804	AP-2 complex subunit alpha-2 (AT5G22770)	185	119	0.00008
isotig28340	AP-1 complex subunit gamma-2 (AT1G60070)	82	45	0.00071
isotig19571	Clathrin adaptor complex small chain (AT5G05010)	14	0	0.00012
isotig13148	Clathrin adaptor complex small chain (AT2G19790)	62	37	0.00879
isotig30868	Putative clathrin assembly protein (AT2G01600)	125	80	0.00133
isotig29605	Putative clathrin assembly protein (AT5G35200)	104	64	0.00155
isotig28671	Phospholipase D alpha 1 (AT3G15730)	39	15	0.00151
isotig29172	E3 ubiquitin-protein ligase COP1 (AT2G32950)	61	34	0.00391
isotig29919	zf-rbx1 (AT3G15070)	164	85	2.80 E^−7^

^a^Data available from *P. notatum* 454/Roche floral transcriptome libraries^31^.

### PN_SCD1 in silico mapping

The possibility that *PN_SCD1* was located within the ACL of *Paspalum* was explored by mapping its sequence *in silico* onto the rice genome and checking the *Paspalum*-rice syntenic relationship of the target regions^19,22^. *PN_SCD1* mapped *in silico* onto a single rice genome site (overlapping gene BGIOSGA001363), placed at chromosome 1 long arm, in segments scattered between positions 24,525,345 and 24,508,528. The total length of the similarity region was 3,494 nt, with E-values ranging between 2.0 e^73^ and 0.023 depending on the length of the fragment aligned. The mapped region was found not syntenic to the ACL^19,22^. Moreover, both *P. simplex* orthologous sequences (PS_6.6 0.1 and PS_6.6.2) mapped *in silico* onto the same rice locus (BGIOSGA001363). No other region showing significant similarity was found in the rice genome. This evidence suggests that *PN_SCD1* is represented by a single copy in the rice genome, that the different sequences detected in both *Paspalum* species are alleles/splice variants of the same gene and that *PN_SCD1* is most probably one of the downstream participants of the molecular cascade involved in the expression of apomixis rather than its trigger. However, we could not assess the representation of *PN_SCD1* directly in the *Paspalum* genome, since a full genome sequence assembly is still not available.

## Discussion

A thorough characterization of the molecular pathways underlying apomixis is a prerequisite for its effective use in breeding. Although in the past few years considerable progress was made on the identification of genes possibly associated to the trait^35^ only one of them (*BABYBOOM*) was able to trigger a specific apomictic step (parthenogenesis) in a sexual background^36^, while no candidate or candidate combination was found capable to govern the whole process involved in the trait. In the genus *Paspalum*, comprehensive lists of candidate genes showing putative positional^22,23^ or expression^24,37^ association with apomixis were produced. However, characterization studies revealed the possible function for only a few of these candidates, like *LORELEI*, *SERK* or *ORC3*^38–40^. Moreover, in spite of all efforts, the triggers of the apospory developmental cascade still remain elusive.

Since an epigenetic control was predicted for some apomixis crucial steps, *i.e*. AI onset^15^, MMC reduction^16^ and parthenogenesis^25^, we focused in the detection of candidate genes showing differential methylation patterns in flowers of apomictic and sexual *P. notatum* genotypes. MSAP was selected as a reference technique to study cytosine methylation in a wide genome basis, due to its simple procedure, low cost and independence on previous sequence characterization. Major methylation polymorphisms were spotted among genotypes, despite a general conservation of methylation proportions. Similar results were found in a prior work dealing with methylation variations in sexual and apomictic *Eragrostis curvula* genotypes^28^. Moreover, an epigenetic similarity clustering analysis revealed that sexual genotypes grouped closer in comparison with apomictic ones. These results are in agreement with a previous work conducted in *P. notatum*, showing that apomictic tetraploids are epigenetically more variable than sexual diploids^29^ and suggest an incomplete and rather disordered cytosine methylation reprogramming occurring in association with apomixis.

Our work confirmed the potential of MSAP used in combination with the Cervera *et al*. matrix construction method^27^ to detect epigenetic regulation differences even when comparing genetically different plants. Naturally, the identification of consistent epigenetic patterns between different reproductive modes turns difficult when genotypes also differ in sequence. But the technique has the potential to detect at least a bias in the occurrence of particular epigenetic features in genetically well conserved sites, even on this highly variable background. Through MSAP analyses involving 547 loci we were able to identify three differentially-methylated sequences expressed in flowers. One of them (PN_6.6) corresponded to a protein coding gene demethylated in apomictic plants and methylated at the internal cytosine in sexual plants. The remaining two sequences (PN_2.10 and PN_8.5) represented an unknown sequence and a functionally not characterized protein coding gene, respectively. However, they both displayed 11/00 MSAP patterns, which disqualified their unequivocal classification as epigenetic marks. Therefore, according to our results, 0.18% of the genome (1 band over 547) unambiguously corresponds to protein coding genes transcribed in flowers and differentially methylated at CCGG sites in apomictic and sexual genotypes. Due to the rather limited MSAP methylation detection capacity (only CCGG sites were surveyed) and the lack of discrimination between full methylation and sequence mutations for 00 patterns, this number likely represents an underestimation. However, our results demonstrated that, even partial, MSAP exploration retains the capacity to contribute to the characterization of the epigenetic status of relevant candidates.

*SCD1* has a reported functional role related with specialized cell division^32^. It encodes the DENN domain and WD repeat-containing protein SCD1, a RAB GTPase- and/or MAPK-interacting protein^32,41^. In the leaf epidermis, the gene participates in the symmetric division of the guard mother cells during stomatal development^32^. *SCD1* has specific RAB GEF activity and it might be involved in the regulation of MAPKs-mediated pathways^32^ as well as the clathrin-mediated membrane transport^33^. Moreover, it was defined as potential substrate receptor of CUL4 E3 ubiquitin ligases^34^. Since RAB-encoding transcripts had been found differentially expressed in sexual and apomictic *Poa pratensis* genotypes^42^, we considered *PN_SCD1* (PN_6.6) worth of further investigation, under the hypothesis that it could represent the second identified member of a RAB-mediated protein localization pathway operative at the onset of apospory. In fact, RNAseq and qRT-PCR analysis revealed *PN_SCD1* upregulation in florets of apomictic plants, in agreement with its de-methylated status. To study further the expression profile, we took advantage of *P. simplex* LCM *Illumina* RNAseq libraries available in our laboratory.

Although *P. notatum* and *P. simplex* belong to different *Paspalum* subgenera (*Anachyris* Nees, Chase for *P. simplex* and *Paspalum sensu stricto* for *P. notatum*), these display almost identical aposporous developmental patterns (*i.e*. several aposporic initials differentiate from nucellar cells giving rise to *i*) pentanucleated embryo sacs lacking antipodals in *P. notatum* and, *ii*) eight-nucleated ones including antipodals in *P. simplex*). The comparison between these two species allows us to identify similar or identical candidate genes between the apomictic and sexual development in two rather different genetic background once the effect of genotype differences have been eliminated by considering several genotypes for each of the two species. After *P. simplex* LCM libraries analyses, *PN_SCD1* was confirmed to be upregulated in the ovule nucellus of apomictic plants at premeiotic stage, suggesting that overexpression is conserved within the genus and might occur at premature developmental stages.

*SCD1* was initially described in plants as a conditional mutant allele that severely disrupted the formation of the cell plate during guard mother cell cytokinesis^32^. The SCD1 protein contains a DENN domain, the distinctive mark of vesicle trafficking regulators, which has been shown to have specific RAB GEF activity^32,41,43^. Moreover, the DENN domain of SCD1 might be involved in the regulation of other signaling routes required for cell expansion and cytokinesis, like MAPKs-mediated pathways^32^. More recently, it was reported that SCD1 functions in clathrin-mediated membrane transport^33^, by associating with isolated clathrin coated vesicles and colocalizing with clathrin light chain 2 in dynamic clathrin-coated pits at or near the plasma membrane^33^.

SCD1 contains also a WD40 domain including a DWD motif, which is defined as the signature of potential substrate receptors of CUL4 E3 ubiquitin ligases^34^, one of the largest subfamilies of CULLIN-RING E3 ubiquitin ligases (CRLs). The enzyme consists of three core subunits: CULLIN4 (CUL4), a RING finger protein REGULATOR OF CULLIN S1 (ROC1)/RING-BOX 1 (RBX1), and UV DAMAGED DNA BINDING PROTEIN1 (DDB1)^44^. While the adapter protein DDB1 assemble the substrate receptor complex, the RING finger protein ROC1/RBX1recruits E2 enzyme to form a catalytic core. Therefore, CUL4 forms a rigid packing architecture for the precise positioning of substrate toward the E2 enzyme, facilitating the ubiquitin transfer^45–47^. As a member of the small subgroup of 85 *Arabidopsis* WD40 proteins containing a DWD motif, SCD1 was reported to act as potential substrate receptor for DDB1-CUL4-ROC1 based E3 ubiquitin ligases^34^. Interestingly, CUL4 is critical for early embryonic development in mice^46^. Bearing this in mind, we decided to evaluate the expression of SCD1 molecular partners (RAB proteins, MAP3Ks, clathrin and CUL4-based E3 ubiquitin ligases and its associated proteins) in the closely related *P. notatum* floral transcriptomes. Interestingly, several of these genes were differentially expressed between apomictic and sexual plants. All of them were upregulated in aposporous plants, suggesting that a vesicle trafficking molecular pathway directing protein localization is operating in particular ways during apomixis, although the path is active in both reproductive modes.

Even when *SCD1* was found demethylated and overexpressed in apomictic plants, the gene is present and active also in sexual genotypes, and even in sexual species. Therefore, the particularity of *SCD1* expression in apomictic plants do not seem to be related with the occurrence of the sequence itself in this particular reproductive type, but with its tissue-specific regulation. Due to its specific expression in the cell layer originating the aposporous initials (*i.e*. the somatic cells acting as progenitors of the aposporous embryo sacs) just before differentiation of these cells, as well as its previously reported role in specialized plant cell division, we hypothesized that *PN_6.6* might be one of the initial components of the molecular cascade associated with aposporous development. We postulate that the upregulation of *PN_SCD1* and its partners in the apospory initials’ precursors might be related with the onset of the cell division processes necessary to generate an unreduced embryo sacs in functionally distinct cell types.

## Methods

### Plant material

The tetraploid (2*n* = 4x = 40) *P. notatum* genotypes used in the MSAP and/or qPCR analysis were selected according to the availability of flowers in the spring/summer season 2015/2016. They were listed below: Q4117 (highly apomictic accession from Brazil, 97% of ovules bearing aposporous embryo sacs, 96% of F1 progenies showing maternal genotypes)^48^; Q4086 (experimental facultative apomictic genotype, 78.4% aposporous embryo sacs, derived from the duplication of the diploid plant Q4084 by colchicine treatment)^49^; C4-4x (sexual tetraploids derived from the duplication of a diploid plant C4-2x)^50^; Q4188 (sexual tetraploid derived from the cross Q3664 x Q3853; parent Q3664 originated from a cross between the sexual tetraploid plant PT-2, induced by colchicine treatment of the sexual diploid Pensacola bahiagrass biotype (*P. notatum* var. *saurae*) and the white-stigma bahiagrass strain WSB^51^; four apomictic (#9, #40, #112, #74) and five sexual (#36, #43, #58, #76, #83) F_1_ hybrids derived from a cross between Q4188 (sexual) x Q4117 (apomictic)^20,21^. A scheme showing the pedigree of the material used is presented in Supplementary Fig. S3.

### MSAP analysis

The MSAP methodology^26^ is based on the use of isoschizomers *Hpa*II and *Msp*I, a pair of restriction enzymes which recognize the same target site (5′-CCGG-3′) but show different sensitivity to the first or second methylated cytosine. *Hpa*II is able to cut when the external cytosine is hemi-methylated (single strand), whereas *Msp*I cuts when the internal cytosine is hemi- or fully methylated (double strand)^27,52,53^. Total genomic DNA was extracted from *P. notatum* inflorescences at anthesis using the method described by Martínez *et al*.^54^. The developmental stages were classified according to the Laspina *et al.*^37^ reproductive calendar. Inflorescences were collected at 9:00 a.m. DNA was used for MSAP analyses as described by Marconi *et al*.^55^. The list of primer combinations used and their relative sequences is provided in Supplementary Table S3. After amplifications, amplified fragments were separated by capillary electrophoresis in an ABI 3130xl Genetic Analyzer (Life Technologies). Comparisons of the patterns produced from samples digested in parallel with *Eco*RI/*Hpa*II and *Eco*RI/*Msp*I allowed identification of genomic alterations in the methylation patterns.

### Epigenetic similarity analysis

To graphically represent the epigenetic landscape in each individual plant, a table was constructed by recording the methylation patterns corresponding to the 547 total loci in all analysed plants. The patterns were codified as I: unmethylated; II: externally methylated; III: internally methylated; IV: fully methylated or absent. Then, we produced a linear graphic representation of the methylation status at each locus for each plant. For clustering analysis, a binary matrix was assembled by codifying the presence of a fragment with 1 and the absence with 0. Then, a second derived binary matrix was produced, codifying the *Hpa*II/*Msp*I amplification patterns as indicated by Cervera *et al*.^27^, in order to represent methylated sites (10 or 01) as 1 and unmethylated or uninformative sites (11 or 00) as 0 (Table 1). After that, a pairwise epigenetic similarity matrix was generated using the Jaccard coefficient^56^. This similarity matrix was used to produce an UPGMA dendrogram.

### Isolation of DNA fragments showing differential methylation

A chi square test was used to classify any biased polymorphism. Therefore, selected samples were run on polyacrylamide gels and silver stained with the aim of isolating and sequencing them as described by Bocchini *et al*.^57^. Recovered polymorphic bands were excised and rehydrated with 100 μl of ultrapure water, by incubating at 4 °C overnight. Samples were centrifuged at maximum speed and the supernatants were transferred into fresh tubes. Five μl aliquots were used as template for re-amplification. They were added to a 20 μl final volume mix containing the same E and M primer combination used in the pre-selective amplification step (Supplementary Table S3). The PCR cycling conditions were: 1 cycle of 94 °C for 1 min, 30 cycles of denaturation at 94 °C for 1 min, annealing at 55 °C for 1 min, extension at 72 °C for 1 min and ending with a 20-min extension step at 72 °C. One μl of the re-amplified DNA was used for cloning into the pCR4-TOPO vector (TOPO TA cloning kit, Invitrogen). After ON culture, plasmids were purified using the GenElute Plasmid Miniprep Kit (Sigma-Aldrich). Both strands were sequenced with the BigDye^®^ Terminator v3.1 Cycle Sequencing Kit (Applied Biosystems) on an ABI 3130xl Genetic Analyzer (Applied Biosystems).

### NGS library resources

Sequences identified through MSAP were used as queries in BLAST searches on *P. notatum* 454/Roche floral transcript databases produced in former studies^31^. Sequences are available at the NCBI Sequence Read Archive database under accession numbers SRX1971037 for apomictic and SRX1971038 for sexual *P. notatum*. The Transcriptome Shotgun Assembly (TSA) projects corresponding to the apomictic (Q4117) and sexual (C4-4x) samples were loaded at DDBJ/ENA/GenBank under the accessions GFMI00000000 and GFNR00000000, respectively. The versions surveyed in this paper are the first ones (GFMI02000000 and GFNR01000000, respectively). Moreover, MSAP fragments were also used for interrogating apomictic and sexual LCM (Laser Capture Microdissection) *P. simplex* Illumina libraries. Both sequences resources were constructed in triplicate from laser microdissected nucellar cells at late premeiosis stage (Galla, Bellucci, Barcaccia and Pupilli, unpublished). Cells were laser-microdissected from the ovule nucellus just before MMC differentiation, as this is the developmental stage immediately preceding the onset of apospory initials in the ovule nucellus of aposporous genotypes^37^.

### Bioinformatic analysis

Annotations were carried out by doing BLASTX at NCBI and confirmed by comparing with results produced by INDEAR (Instituto de Agrobiotecnología de Rosario, Argentina) using TRINOTATE (https://trinotate.github.io/). *In silico* mapping predictions were conducted by doing BLASTN searches onto the *Oryza sativa* subsp. Indica genome at the Gramene website (www.gramene.org). For 454/Roche comparative quantitation, we used the data originated from mapping the reads originated from each 454 library (apo or sex) onto a global (apo + sex) assembly^31^. For quantification analyses onto the *P. simplex* Illumina LCM libraries, the isotigs identified in the *P. notatum* 454/Roche library were used as queries in BLASTN and BLASTX surveys, and statistically significant differential expression was detected by applying Baggerly tests^58^. PANTHER Gene Ontology (GO) Term Enrichment Analysis (http://pantherdb.org) was used to detect overrepresentation of Single Organism Transport and Localization GO biological processes in the *P. notatum* 454/Roche libraries^59,60^.

### RNA isolation and cDNA synthesis

Florets at anthesis were collected at 9:00 a.m and classified according to the method recommended by Laspina *et al*.^37^. Total RNA samples were extracted by using the SV Total RNA Isolation System (Promega, Madison, WI, USA), according to the manufacturers’ protocol. Total RNA concentrations and quality indexes were determined by spectrophotometry at 260/280 nm. RNA was retro-transcribed using SuperScript^TM^ II Reverse Transcriptase (Invitrogen Life Tecnologies, California, CA, USA). Briefly, 1 μg RNA, 10 pmol oligo-dT and 1 μl dNTP mix (10 mM each) were mixed in a 12 μl final volume. After 5 min of 65 °C incubation, 4 μl of 5X First-Stand Buffer and 2 μl 0.1 M DTT were added to the mix. All components were incubated at 42 °C for 2 min. Then, 1 μl of SuperScript RT II was added. The reaction was incubated for 50 min at 42 °C and then stopped by heating at 70 °C for 15 min. The cDNA samples were stored at −80 °C until use.

### Quantitative RT-PCR analysis

Gene-specific PCR primer pairs were designed by using Primer3 v.0.4.0 (http://biotools.umassmed.edu/bioapps/primer3_www.cgi)^61^ (Supplementary Table S4). Oligonucleotides were provided by GBT (GeneBiotech, Argentina). RT-PCR reactions were prepared in a 25 µL final volume reaction containing 200 nM gene specific primers, 1X Mezcla Real qPCR (Biodynamics, Argentina) and 20 ng of reverse-transcribed RNA. Beta-tubulin was used as normalizer gene, since it was characterized as an optimal reference in analogous *P. notatum* apomictic vs. sexual systems^38,39^. RT (-) and non-template controls were incorporated to the assays. Each biological replicate was processed including three technical replicates. Amplifications were performed in a Rotor-Gene Q thermocycler (Qiagen), programmed as follows: 2 min at 94 °C, 45 cycles of 15 s at 94 °C, 30 s at 57 °C, 40 s at 72 °C. Melting curves (10 s cycles from 72 to 95 °C, where temperature was increased by 0.2 °C after cycle 2) were produced at the end of the cycling to check consistent amplification of a single amplicon. Relative quantitative expression was estimated by using REST-RG (Relative Expression Software Tool V 2.0.7 for Rotor Gene, Corbett Life Sciences), considering the take-off values and amplification efficiencies for each particular reaction. The REST9 software allows relative quantitation by making comparisons between samples in pairs. Sexual plant C4-4x was used as control, referring the expression of each sample to this particular genotype.

### Availability of materials and data

The plant materials used as progenitors in the current study were collected/developed and characterized by Prof. Camilo L. Quarin at Instituto de Botánica del Nordeste (IBONE), CONICET-UNNE, Corrientes, Argentina and described in prior works of our research group^48,50,51^. All materials belong to the IBONE’s live germplasm collection. Plants were grown in accordance with the local legislation at IBONE-CONICET-UNNE and IICAR-CONICET-UNR, Rosario, Argentina. Voucher specimens of this material are deposited at the Herbarium CTES-IBONE (publicly available), under deposition numbers: C4-4X (Quarin, C. L. 4260, barcode CTES0541627, cardboard No. 330064); Q4188 (Quarín, C. L. 4188, barcode CTES0542168, cardboard No. 323424; Q4117 (Quarin, C. L. 4117, barcode CTES0541626, cardboard No. 233851); Q4086 (Quarin, C. L. 4086, barcode CTES0542167, cardboard No. 219629). Part of the evidence presented here was retrieved from datasets reported in a prior article^31^, which are publicly available at: NCBI Sequence Read Archive database, accession numbers SRX1971037 and SRX1971038; Transcriptome Shotgun Assembly (TSA) projects, DDBJ/ENA/GenBank, accessions GFMI00000000 and GFNR00000000 (the versions surveyed in this paper are the first ones, GFMI02000000 and GFNR01000000, respectively).

## Electronic supplementary material

Supplementary materials
