# Supplementary material for: The vesicle trafficking regulator PN_SCD1 is demethylated and overexpressed in florets of apomictic Paspalum notatum genotypes
Source: Sci Rep. 2018 Feb 14;8:3030. doi: 10.1038/s41598-018-21220-4 (PMC5812994; doi:10.1038/s41598-018-21220-4)
Supplement: Supplementary file 1 — Supplementary materials [file 41598_2018_21220_MOESM1_ESM.pdf]

Supplementary Information

The vesicle trafficking regulator *PN_SCD1* is demethylated and overexpressed in florets of apomictic *Paspalum notatum* genotypes

Marika Bocchini, Giulio Galla, Fulvio Pupilli, Michele Bellucci, Gianni Barcaccia, Juan Pablo A. Ortiz, Silvina C. Pessino and Emidio Albertini*

*correspondence to:
Emidio Albertini, emidio.albertini@unipg.it

Supplementary Fig. S1. Tree plot representing epigenetic similarities among sexual and apomictic genotypes. The clustering was based on a similarity matrix reflecting epigenetic distances. While sexual plants grouped together, apomictic ones showed a rather dispersed pattern, revealing a less organized cytosine methylation distribution.

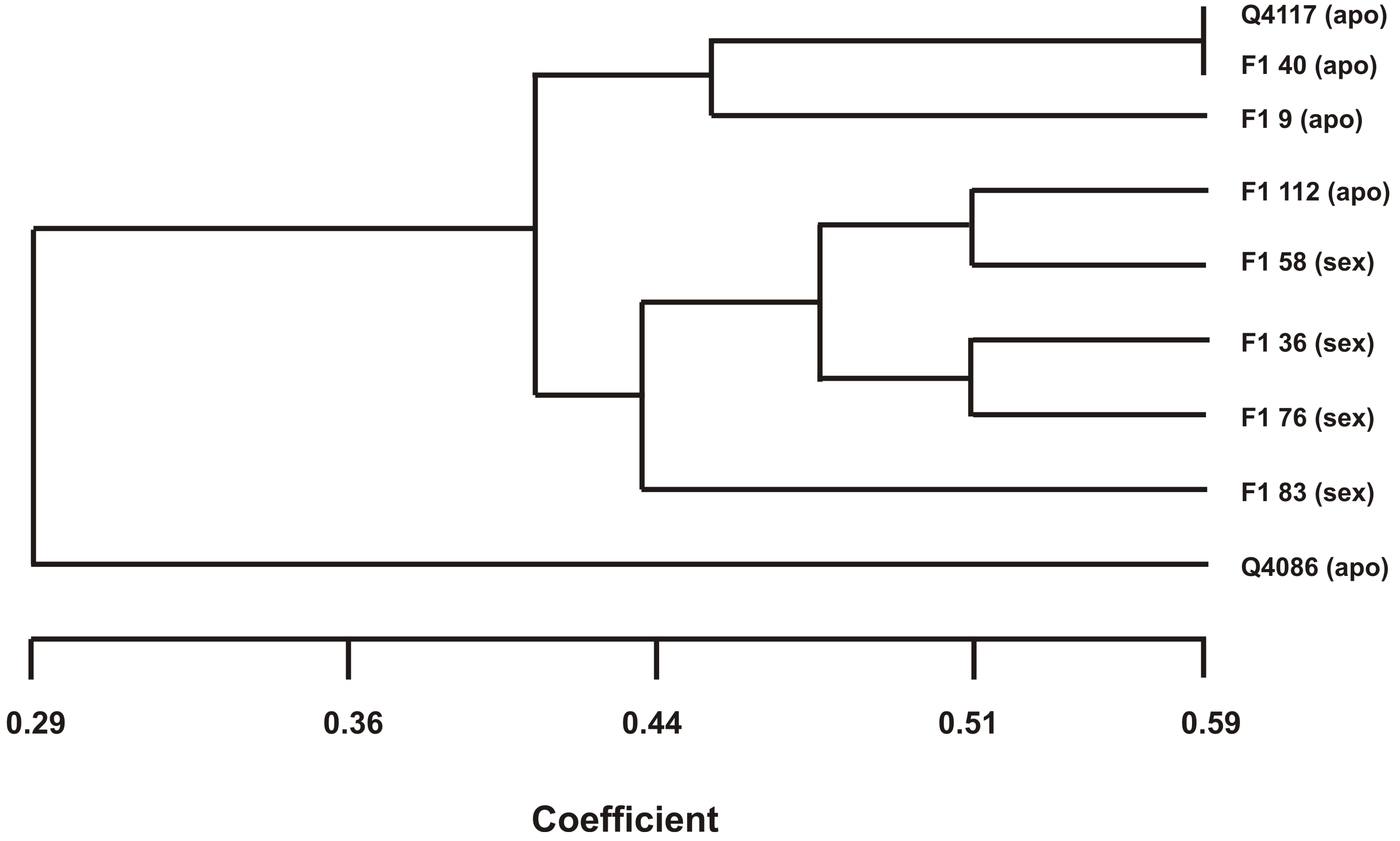

Supplementary Fig. S2. Matrix showing the bands displaying a trend to differential methylation between sexual and apomictic plants. 1: presence of the band; 0: absence of the band. The primer combination for each polymorphic band is reported on the second column and is indicated as in Table S3. Figure also gives information on the molecular weight of each polymorphic band (MW) and the name chosen for selected polymorphic bands.

			Hpa II					Msp I					Hpa II				Msp I			
			apo	apo	apo	apo	apo	apo	apo	apo	apo	apo	sex	sex	sex	sex	sex	sex	sex	sex
			19/37/55	20/38/56	21/39/57	22/40/58	23/41/59	28/46/64	29/47/65	30/48/66	31/49/67	32/50/68	24/42/60	25/43/61	26/44/62	27/45/63	33/51/69	34/52/70	35/53/71	36/54/72
name	primer comb.	MW	1	2	3	4	5	10	11	12	13	14	6	7	8	9	15	16	17	18
	Comb_1	249,9	1	0	1	1	0	1	0	1	1	1	0	0	0	0	0	1	0	0
PN_1.7	Comb_2	126,7	1	1	1	1	0	1	1	1	1	0	0	0	0	0	0	0	0	0
PN_2.10	Comb_2	127,8	1	1	1	1	0	1	1	1	1	0	0	0	0	0	0	0	0	0
PN_1.8	Comb_2	203,5	1	1	1	0	0	1	1	1	0	0	0	0	0	0	0	0	0	0
PN_2.2	Comb_2	213,1	1	1	1	1	0	1	1	1	0	0	0	0	0	0	0	0	0	0
	Comb_2	228,4	1	1	1	1	0	1	1	0	0	0	0	0	0	0	0	0	0	0
	Comb_2	334,1	1	1	1	1	0	1	0	1	0	0	0	0	0	0	0	0	0	0
PN_4.8	Comb_3	317,4	1	1	1	1	0	1	1	1	1	0	0	0	0	0	0	0	0	0
	Comb_4	55,7	0	0	0	0	0	0	1	1	1	1	0	0	0	0	1	0	0	0
PN_4.10	Comb_4	130,4	0	1	0	0	0	0	1	0	0	0	0	1	1	1	1	1	1	1
	Comb_5	51	1	0	1	1	1	1	1	1	1	1	0	1	0	0	1	0	0	0
	Comb_5	159,7	0	0	0	0	1	0	0	0	0	1	1	1	1	1	0	1	1	1
	Comb_5	160,8	0	0	0	0	0	0	0	0	0	0	0	1	1	1	0	0	0	1
	Comb_6	59,2	0	0	0	0	0	1	1	1	1	0	0	0	0	0	0	0	0	0
PN_6.6	Comb_6	78,2	0	1	1	1	1	1	1	1	1	1	0	0	0	0	1	1	1	1
	Comb_7	191,5	1	0	1	0	1	1	1	1	0	0	0	0	0	0	0	0	0	0
PN_6.5	Comb_7	319,6	1	1	0	1	1	1	1	0	1	1	0	1	0	0	0	0	0	0
PN_7.6	Comb_8	124,2	1	0	1	1	1	1	0	1	1	1	0	0	0	0	0	0	0	0
PN_8.5	Comb_8	289	1	1	1	1	0	1	1	1	1	0	0	0	0	0	0	0	0	0
	Comb_9	101,4	0	0	0	0	0	0	0	1	0	0	0	0	0	1	1	1	1	1
	Comb_10	173,1	0	0	0	0	1	0	0	0	0	0	1	1	1	1	1	1	0	1
PN_7.7	Comb_10	239,9	1	1	1	1	1	1	1	1	1	0	1	1	1	1	0	0	0	0
	Comb_11	181,4	0	0	1	0	0	0	0	1	0	0	0	1	1	0	1	1	1	1
	Comb_11	235	0	1	1	0	1	0	1	1	0	1	0	0	0	0	0	0	0	0
	Comb_11	399,3	0	0	0	0	1	0	0	0	0	1	1	0	1	1	1	0	1	1
PN_8.8	Comb_11	449,6	0	0	0	0	0	0	0	0	0	0	1	1	1	1	1	1	1	1

Supplementary Fig. S3. Scheme representing the pedigree of the genotypes used in the MSAP and qPCR analysis. Red: genotypes used in MSAP analysis only. Blue: genotypes used in qPCR analysis only. Green: genotypes used in both MSAP and qPCR experiments. Genotype Q4188 was used as pistillate parent to generate the F1 hybrids, but it was not used in MSAP or qPCR assays.

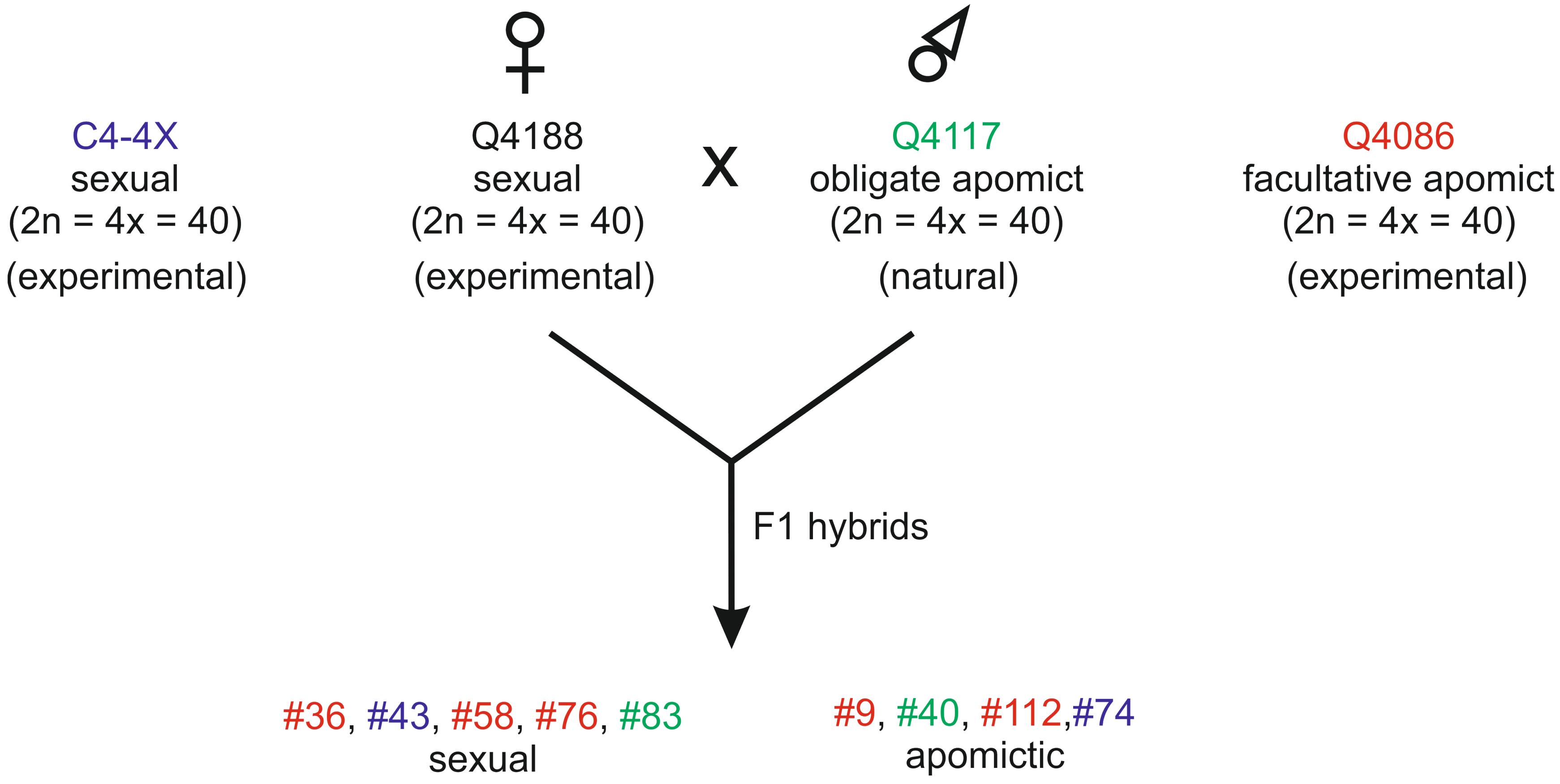

Supplementary Table S1: *In silico* mapping of 12 polymorphic MSAP fragments onto the *Oryza sativa* Indica genome

MSAP fragment	Rice chromosome (best match)	Location	Alignement (bp)	E-value	Gene
PN_1.7	2	5839850 to 5839867 (-)	18	0.6	-
PN_1.8	2	5839850 to 5839867	18	0.88	-
PN_2.2	3	30661403 to 30661419 (+)	17	0.11	-
PN_2.10	8	15896250 to 15896268 (-)	19	0.23	-
PN_4.8	1	9033199 to 9033216 (-)	18	0.83	-
PN_4.10	5	9607518 to 9607540 (+)	23	0.26	-
PN_6.5	10	20316442 to 20316459 (+)	18	0.51	BGIOSGA033396 Putative uncharacterized protein
PN_6.6	1	24511091 to 24511144 (+)	54	$1.9E^{-15}$	BGIOSGA001363 SCD1-like
PN_7.6	11	4874377 to 4874393 (+)	17	1.8	BGIOSGA034339 Putative uncharacterized protein
PN_7.7	11	4874377 to 4874393 (+)	17	1.8	BGIOSGA034339 Putative uncharacterized protein
PN_8.5	11	4681749 to 4681766 (-)	18	0.66	-
PN_8.8	9	18652083 to 18652103 (-)	21	0.019	-

Supplementary Table S2. qPCR analysis reveals up-regulation of PN_6.6 in florets of apomictic plants

Plants[a]	Relative expression[b]	Differential regulation prediction[b]	Standard error[b]
C4-4xS vs C4-4xS	1	-	-
C4-4xS vs 43S	1.339	-	0.889- 2.663
C4-4xS vs 83S	1.228	-	0.674- 2.729
C4-4xS vs 40A	3.997	UP	2.815- 7.113
C4-4xS vs 74A	6.030	UP	2.859- 10.634
C4-4xS vs Q4117A	6.827	UP	4.142- 14.277

[a] Sample pairs compared with the REST-RG program (Relative Expression Software Tool V 2.0.7 for Rotor Gene, Corbett Life Sciences). Expression was referred to control plant C4-4x. The letter placed after each plant identifier indicates the reproductive mode: S: sexual; A: apomictic.
[b] Relative expression values, regulation prediction and standard errors were calculated by the REST-RG software.

Supplementary Table S3. Adapters and primers used in MSAP experiments

**Adapter or Primer**	***EcoR*I (5'-3')**	***Hpa*II / *Msp*I (5'-3')**
Adapter 1[a]	CTCGTAGACTGCGTACC	GATCATGAGTCCTGCT
Adapter 2[a]	AATTGGTACGCAGTCTAC	CGAGCAGGACTCATGA
Pre-amplification primers	E: GACTGCGTACCAATTC+C	H/M: GATGAGTCTAGAACGG+A
Selective amplification primers		
comb_1	E+CAC	H/M+ATC
comb_2	E+CAC	H/M+ACT
comb_3	E+CAC	H/M+AAT
comb_4	E+CAG	H/M+ATC
comb_5	E+CAG	H/M+AAT
comb_6	E+CAG	H/M+ACT
comb_7	E+CCA	H/M+AAT
comb_8	E+CCA	H/M+ACT
comb_9	E+CCA	H/M+ATC
comb_10	E+CAA	H/M+AAT
comb_11	E+CAA	H/M+ACT
comb_12	E+CAA	H/M+ATC

[a]Adapter 1 and 2 are the complementary single strands used in combination to produce the double strand adapter.

Supplementary Table S4. Primers used in qPCR experiments

Primer	Sequence (5'-3')	Length of the amplified fragment	TM (°C)
PN_6.6 for	GCTGGATTGGGATTACCTGA	119	59.89
PN_6.6 rev	TGTTGCAGATGTGTGAGCAA		60.03
ß-tubulin for	GTGGAGTGGATCCCCAACAA	158	63.23
ß-tubulin rev	AAAGCCTTCCTCCTGAACATGG		65.85
